# Highly Stable InSe-FET Biosensor for Ultra-Sensitive Detection of Breast Cancer Biomarker CA125

**DOI:** 10.3390/bios13020193

**Published:** 2023-01-28

**Authors:** Hao Ji, Zhenhua Wang, Shun Wang, Chao Wang, Kai Zhang, Yu Zhang, Lin Han

**Affiliations:** 1 Institute of Marine Science and Technology, Shandong University, Qingdao 266237, China; 2 Shenzhen Research Institute of Shandong University, Shenzhen 518057, China; 3 Shandong Engineering Research Center of Biomarker and Artificial Intelligence Application, Ji’nan 250100, China

**Keywords:** field-effect transistor, InSe, biosensor, CA125, biomarker detection, liquid gate

## Abstract

Two-dimensional materials-based field-effect transistors (FETs) are promising biosensors because of their outstanding electrical properties, tunable band gap, high specific surface area, label-free detection, and potential miniaturization for portable diagnostic products. However, it is crucial for FET biosensors to have a high electrical performance and stability degradation in liquid environments for their practical application. Here, a high-performance InSe-FET biosensor is developed and demonstrated for the detection of the CA125 biomarker in clinical samples. The InSe-FET is integrated with a homemade microfluidic channel, exhibiting good electrical stability during the liquid channel process because of the passivation effect on the InSe channel. The InSe-FET biosensor is capable of the quantitative detection of the CA125 biomarker in breast cancer in the range of 0.01–1000 U/mL, with a detection time of 20 min. This work provides a universal detection tool for protein biomarker sensing. The detection results of the clinical samples demonstrate its promising application in early screenings of major diseases.

## 1. Introduction

Biomarkers play a key role in disease diagnosis and treatment [1,2,3]. The early detection of major diseases such as cancer can be achieved by detecting biomarkers in the blood. Various immunoassays have been proposed and investigated to meet the growing demand for assays while ensuring sensitivity and specificity [4,5]. As an example, fluorescent immunoassays can provide highly sensitive and reliable detection in aqueous or cellular environments, but their cumbersome steps cause a reduction in efficiency [6]. In recent years, field-effect transistor (FET) biosensors, as a promising label-free and fast biomolecular detection method, have attracted much attention due to their low power consumption, scalability to on-chip integration, and low processing cost [7,8,9,10]. Biosensors constructed with various nanomaterials and nanostructures, including silicon nanowires and carbon nanotubes, have shown potential in improving the detection sensitivity of biomolecules [11,12,13,14,15]. In addition, two-dimensional (2D) nanomaterials, such as graphene and transition metal sulfide (TMD) compounds, show ultrasensitive properties for existing detection methods. The active layer of the 2D material allows for a highly specific surface area, resulting in superior charge sensitivity [16,17,18]. In particular, unlike zero-bandgap graphene, the presence of a bandgap in TMDs is critical for FET-based platforms because the modulation of two-dimensional channel carrier transport is triggered by binding processes between the surface and biomolecules [19]. Recently, MoS_2_-based FETs have been used for the immediate diagnosis of nucleic acid molecules and biomolecules, such as proteins, demonstrating the potential of two-dimensional semiconductor materials for applications [20,21].

For FET-based biosensors, a liquid gate (Ag/AgCl reference electrode) is commonly used. Liquid gate modulated FETs with low power consumption and easy detection are widely used in graphene- and MoS_2_-based FET biosensors. However, in ionic liquids, the liquid gate modulation process is similar to that of an electrochemical reaction, and the operating voltage is prone to the electrolysis of the aqueous solution, causing the decomposition of the channel material. Specifically, some potential 2D materials for biomolecular sensing require further evaluation of the impact of liquid gate modulation. High-mobility 2D materials have natural advantages in sensor construction and can achieve highly sensitive detection [22]. As a III–VI two-dimensional semiconductor compound, InSe has a direct and moderate band gap of 1.26 eV with an ultra-high Hall mobility of over 1000 cm^2^ V^−1^ s^−1^ at room temperature due to the light electron effective mass (∼0.143 m_0_), making it an ideal material for biomolecular sensing [23].

In this study, we propose an InSe-FET biosensor and investigate its electrical stability in the liquid channel. In addition to the commonly used liquid gate electrode, the back gate working mode is applied to conduct the biomarker detection. The liquid channel works as a passivation layer and effectively improves the stability of InSe-FETs, even though the field-effect mobility degrades slightly due to the scattering of ions in the liquid channel. The proposed InSe-FET biosensor achieves an extra-large quantitative linear and selective detection range during CA125 biomarker sensing. The detection capability of the CA125 biomarker in clinical serum samples demonstrates its potential applications in the screening of major diseases.

## 2. Materials and Methods

### 2.1. Materials and Regents

Bulk InSe was bought from XFNANO Materials Tech Co., Ltd., Shenzhen, China. Silicon wafers were purchased from Saibang Electronic Technology Co. Ltd., Kunshan, China. Phosphate-buffered saline (PBS) was purchased from Corning. 3-aminopropyltrimethoxisylane (APTES, >99%) was purchased from Sigma-Aldrich. Capture antibodies, recombinant proteins, and detection antibodies, CA125 and CA199, were purchased from Fitzgerald (America). The types and purity of all antigens and antibodies used in the experiments are summarized in Table 1. Clinical serum specimens were collected at Qilu Hospital of Shandong University. The ultrapure water (18.25 MU/cm^3^) used throughout all experiments was made by a Millipore system. All chemicals used in this work were of analytical grade or highest purity available and used directly without further purification. Acetone (>99.9%) and isopropyl alcohol (>99.7%) were purchased from Sinopharm.

### 2.2. Clinical Samples Preparation

First, 1–2 mL of venous blood from healthy person and patient is collected in a non-anticoagulant tube and is kept standing for 2 h. Then, the supernatant is transferred into a new Eppendorf tube and centrifuged at 3000 rpm for 10 min. Finally, 100 μL of serum supernatant is collected and stored at −80 or −20 °C for testing.

### 2.3. InSe Material Characterization

InSe is examined using high-resolution transmission electron microscope (TEM) (FEI-G20, Thermo Fisher Scientific Inc., Waltham, MA, USA). The thickness of the InSe is determined using atomic force microscopy (AFM) on a Smart SPM AFM system. Raman spectra and photoluminescence of InSe are determined by a Renishaw inVia Raman microscope at room temperature with a 532 nm laser as an excitation source.

### 2.4. Fabrication of InSe-FET-Based Biosensor

**Figure 1:**
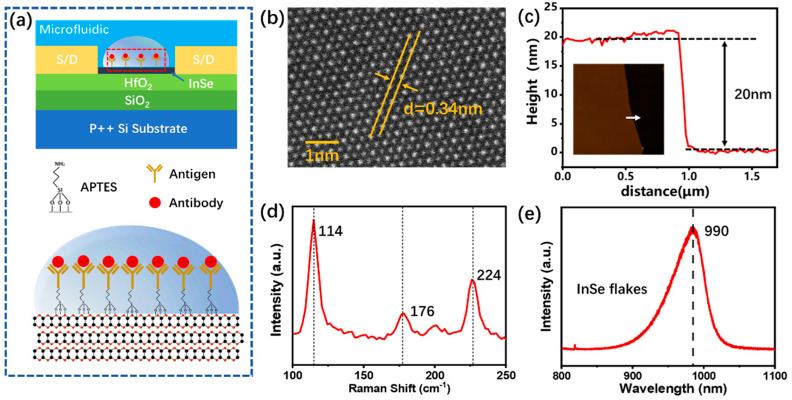
(**a**) Schematic of the InSe-FET biosensor for biomarker CA125 detection. (**b**) High-resolution transmission electron microscopy of InSe. (**c**) Thickness of typical InSe films used in FET determined by AFM. (**d**,**e**) Raman spectra and photoluminescence spectra of transferred InSe flakes.

A schematic diagram of the InSe-FET biosensor fabrication process is shown in Figure S1. The p-doped Si substrate with 100 nm SiO_2_ is cleaned using acetone, methanol, and deionized water. Then a high-quality layer of HfO_2_ is grown on the Si/SiO_2_ substrate by thermal ALD (atomic layer deposition) as a way to reduce the scattering centers generated by fixed groups and defects on the SiO_2_ surface [24,25]. The PE-ALD Beneq TFS200 Instrument is used for the deposition of HfO_2_ films from tetrakis-dimethylamino hafnium (TDMAHf) precursor. The TDMAHf is heated at 75 °C and N_2_ carrier gas is flown at 40 sccm to improve the hafnium precursor transport from the bubbler to the reactor chamber. The oxidation step is performed using water vapor in case of the thermal ALD at 250 °C, while the number of cycles ranges from 125 (growth rate is about 0.08 nm/cycle) for the T-ALD process. The InSe with few layers is transferred from the bulk material to the substrate by mechanical exfoliation. Ti/Au (10 nm/20 nm) source/drain electrode is formed by high-vacuum (4 × 10^−5^ Pa) electron beam evaporation using a shadow mask to avoid the introduction of contamination by the photoresist, creating a good ohmic contact. The linearity of the output curve in Figure S2 illustrates the good ohmic contact of the device at room temperature. A microfluidic chip with a channel of approximately 5000 × 40 × 20 μm^3^ (the width of the flow channel is 20 μm, the height is 50 μm, and the length is 5000 μm) is designed and aligned with the InSe sensing channel to form a biosensor, allowing solutions to flow into the channel and interact with the InSe channel. The dimensions of the PDMS device are 10 × 5 × 2 mm^3^ (see in Figure S3). The schematic cross-sectional structure of the InSe biosensor is shown in Figure 1a. The channel current can be regulated by the voltage applied to the back gate or liquid gate (Ag/AgCl reference electrode) mode. The electrical performance and sensing tests are performed using a Keithley 2636B system for I–V testing. The fabrication process’ time consumption schematic graphs and photograph of fabricated InSe-FET biosensor are shown in Figure S3.

### 2.5. Capture Antibody-CA125 Immobilization and CA125 Antigen Sensing

APTES solution is made of 0.4 mL APTES with ethanol and DI water (19 mL/1 mL), and then injected into the flow channel and incubated with InSe for 1 h. After incubation, InSe channel is cleaned by inflowing ethanol and DI water. A concentration of 100 ug/L antibody-CA125 is loaded into the microfluidic channel and incubated for 2 h to achieve adequate immobilization on InSe channel. After cleaning, CA125 antigen sample is loaded in the detection channel and the reaction time is 20 min. Finally, the output current is detected after cleaning of reaction residues using 1 × PBS.

### 2.6. Electrical Characterization of InSe-FET

**Figure 2:**
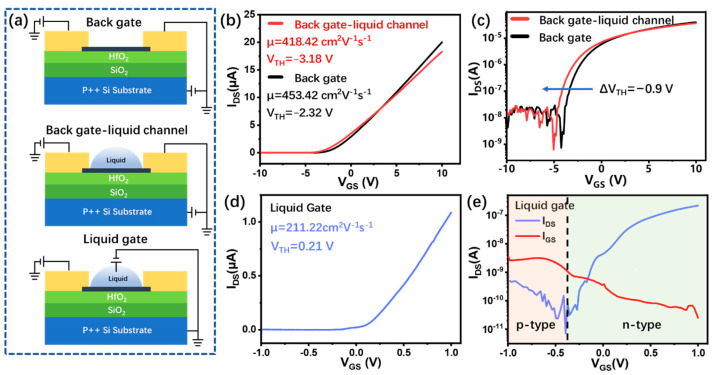
(**a**) Schematic diagram of the three operating modes of InSe-FET. Comparison of InSe-FET performance before and after filling the microfluidic channel with liquid in (**b**) linear and (**c**) logarithmic coordinates. Transfer characteristic curves of InSe-FET regulated by liquid gate in (**d**) linear and (**e**) logarithmic coordinate systems.

The I_DS_−V_DS_ output characteristics and I_DS_−V_GS_ transfer characteristics of the InSe-FET are measured by a Keithley 2636 under ambient and liquid conditions. The working mode includes back gate and liquid gate modes. For the back gate mode, V_BG_ is swept from −10 V to 10 V at V_DS_ = 0.1 V to obtain transfer characteristic. For liquid gate, V_LG_ is swept from −1 V to 1 V at V_DS_ = 0.1 V. The details of circuit connections and the experimental setup are shown in Figure 2a. For the InSe-FET stability test, the same parameters as for the electrical test are selected, and the transfer curves are tested at 15 min intervals under atmospheric and liquid environments. The transfer characteristics are measured for the fabricated InSe-FET biosensor after antibody immobilization and after CA125 capture antibody immobilization, respectively.

## 3. Results

### 3.1. Characterization of the InSe

The schematic structure of the InSe-FET biosensor is shown in Figure 1a. The back gate InSe-FET is integrated with the homemade microfluidic channel. High-quality ALD-grown HfO_2_ is applied to effectively improve field-effect mobility and reduce hysteresis by reducing the parasitic capacitance and shielding the interfacial Coulomb scattering [26]. As shown in Figure S4, the hysteresis of the transistor with the HfO_2_/SiO_2_ substrate is about 0.5 V, which is significantly less than its value, 3.8 V, with the SiO_2_ substrate only. APTES as a coupling agent is functionalized directly on the InSe surface by the chemical reaction of C-Si bonds and Se vacancy defects [27]. Although the InSe lacks suspension bonds, the highly reactive sites in the Se vacancies can react with APTES to form In-O bonds [28]. The atomic force microscopy characterization in Figure S5 demonstrates that the surface roughness Ra increases from 0.1264 nm to 0.5062 nm after the APTES functionalization on InSe. In order to confirm the successful functionalization of APTES and the immobilization of anti-CA125, Raman spectroscopy was conducted after anti-CA125 immobilization on APTES-modified InSe in comparison to that on bare InSe. A random spot in the channel and the channel area’s scanned Raman spectra are shown in Figure S6. They show representative peaks of anti-CA125 at 1096 cm^−1^ and 1407 cm^−1^ on the APTES-modified InSe channel, and the scanned Raman spectra present a uniform distribution of anti-CA125, indicating the successful functionalization of APTES and the immobilization of anti-CA125. APTES is functionalized directly on the InSe surface as a coupling agent without an additional top passivation structure, allowing the surface charge of the detection antigen to act directly on the channel surface to introduce changes in output current, effectively improving the detection sensitivity [29]. The electrical properties of the FET devices are determined by the quality of the InSe films, which strongly influences the sensing performance of the biosensors. In order to characterize the quality of the InSe material, the atomic structure of the InSe is determined by high-resolution electron scanning transmission microscopy as shown in Figure 1b. The multilayered InSe shows a complete honeycomb structure with alternating rows of In and Se atoms with different brightnesses corresponding to different atomic numbers. The atomic structure indicates that the InSe used in the experiment is in the γ-conformation with the lattice constant a = b = 0.34 nm, which is consistent with previous reports [30]. It has been shown that a higher device performance and smaller thickness of the material are beneficial in improving the sensitivity of the sensor [31]. FETs constructed with 20–30 nm thick InSe have the best performance [22,26] and are conducive to improving the device response to the biosensor. Thus, an InSe film with a thickness of about 20 nm is selected to construct the InSe-FET, and the thickness conducted by AFM is shown in Figure 1c. As shown in Figure 1d, three peaks can be observed at 114 cm^−1^, 176 cm^−1^, and 226 cm^−1^ for the InSe layers, which are consistent with the Raman modes of γ-InSe [32]. The band gap of the transferred InSe can be inferred from the photoluminescence (PL) spectrum in Figure 1e, and the peak at 990 nm corresponding to the band gap of the multilayer InSe is calculated to be about 1.25 eV. The typical multilayer InSe material properties are presented by the Raman and PL spectra of the InSe, indicating the successful transfer of InSe flakes to the substrate.

### 3.2. Electrical Characterization of InSe-FET

The working mode of InSe-FET plays an essential role in biosensing performance. Here, the performances of the InSe-FETs working in the back gate liquid channel and top liquid gate are compared with those of the fabricated InSe-FET. As shown in Figure 2a, the fabricated device tested in the air (dry channel) is used as a benchmark to evaluate the difference in the electrical performance between the two working modes in a liquid environment. Figure 2b and c shows the transfer characteristics of the InSe-FET working in the back gate liquid channel on linear and logarithmic scales in comparison with the fabricated InSe-FET, and the slight decrease in the slope implies a slight decrease in the field-effect mobility from 453.42 to 418.42 cm^2^/V^−1^s^−1^, which may be caused by the adsorption of the ion group in the PBS solution on the InSe surface. The field-effect mobility of multilayer InSe-FETs can be extracted from the transfer curve using the following equation: μ = [L/WC_i_V_DS_] × [dI_DS_/dV_G_], where L is channel length of 30 μm, W is the channel width of 20 μm, and Ci is the series capacitance of 100 nm SiO_2_ and 10 nm HfO_2_, where the dielectric constants of both are 3.9 and 15, respectively. The negative shift of −0.9 V in the threshold voltage in the logarithmic coordinate system in Figure 2c indicates that the InSe-FET appears to be n-doping in the PBS solution [19], which is possibly due to the charge transfer of the OH-groups on the InSe surface [6]. The InSe-FET in the liquid gate working mode switches between the on and off states in the 2 V range with a higher regulation efficiency compared with that of the back gate mode, as shown in Figure 2d. However, a calculated mobility of 211.22 cm^2^/V^−1^s^−1^ is obtained, which decreases by 50% compared to that of the back gate working mode. The larger mobility drop is probably due to the smaller electron effective mass of InSe, which is exceptionally sensitive to interfacial Coulomb scattering [26]. In the liquid gate working mode, it forms a 2~3 nm charge distribution layer at the InSe–liquid interface, as shown in Figure S7, in which the thickness range of the induced charges is only due to the direct formation of a double electric layer on the surface of InSe by liquid gate regulation [33]. The formation of a bilayer on the InSe surface by the liquid gate modulates the channel current while increasing the scattering chance of the induced charge near the surface of the InSe. Figure 2e shows that the device has clearly ambipolar behavior in the liquid gate working mode. A similar phenomenon was previously observed in liquid gate MoS_2_ layers of more than 10 nm [30], but this is the first time that it has been found in the InSe materials. This indicates that the occurrence of ambipolar phenomena is most likely related to the efficient modulation of the charge transport properties of the InSe surface layer by the liquid gate and not the intrinsic properties of the InSe material. The gate leakage current increases when the liquid gate bias voltage is negative, as shown in Figure 2e. The basic FET performance indicates that the back gate working mode is appropriate for biomarker sensing.

### 3.3. Electrical Stability of InSe-FET 

**Figure 3:**
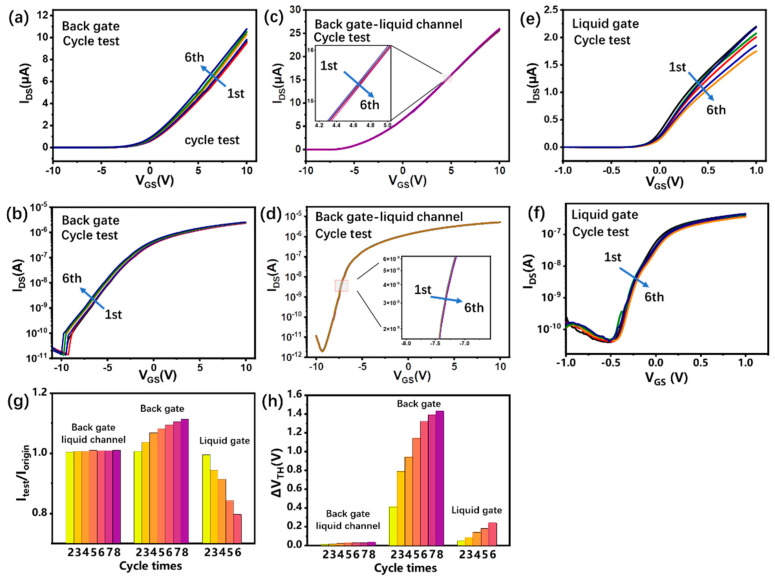
Results of cyclic tests in three modes. The transfer characteristics cycle tests of InSe-FET in (**a**,**b**) back gate dry channel, (**c**,**d**) back gate liquid channel, (**e**,**f**) and liquid gate in linear and logarithmic coordinates. The current (**g**) and threshold voltage (**h**) evolution of three modes with cycle times.Note: In back gate working mode, the point at V_DS_ = 0.1 V, V_GS_ = 10 V is selected to compare output current change and threshold shift; in liquid gate working mode, the point at V_DS_ = 0.1 V, V_GS_ = 1 V is selected to compare output current change and threshold shift.

During biomarker detection, the electrical stability during the test cycles determines the reliability and sensitivity of the InSe-FET biosensor. Here, in order to test the electrical stability, the transfer characteristics are scanned eight times at an interval of 15 min for the InSe-FETs in the different working modes. However, the InSe-FETs usually fail on the seventh test. Figure 3a and b shows the transfer curves of the fabricated InSe-FET in the air corresponding to the linear and logarithmic coordinates. As the number of tests increases, the output current slightly drops and threshold voltage drifts are observed, which are probably due to the desorption of water and oxygen on the surface of the InSe in the atmosphere. This non-stationarity is more pronounced in the first few tests of the freshly processed device, indicating the rapid desorption of water and oxygen on the InSe surface in the atmosphere. When the devices are tested in a liquid environment through back gate modulation, an extremely high stability was observed, as shown in Figure 3c,d, and there were no observable changes in the transfer curves over the eight repeating tests. The liquid in the flow channel actually plays a similar role to passivation, avoiding the creation of unstable states caused by adsorbed substances on the InSe surface. The excellent electrical stability is especially important for biosensors for ultra-low-concentration biomolecule detection [31]. When the InSe-FET is working with the liquid gate, the transfer curve shows a significant drop in current in several of the repeating tests and is irrecoverable. This irreversible property degradation is probably due to the change in material properties induced by the liquid electrode. Due to the reactivity of InSe [28], an electrochemical etching-like reaction probably occurs on the InSe surface, causing irreversible electrical property degradation of the device, even if the voltage applied to the liquid gate is small. Figure 3g shows the negligible change in output current for the InSe-FET in the back gate and liquid channel working modes. The output current increased 1.11 times after eight replications for InSe-FET in the back gate and dry channel working modes, while the output current decreased to 0.79 after six replications for the top liquid gate working mode. Figure 3h shows slight threshold voltage shifts of 0.032 V after eight scans for InSe-FET in the back gate and liquid channel working modes, positive threshold voltage shifts up to 1.43 V after eight scans for InSe-FET in the back gate and dry channel working modes, and positive threshold voltage shifts up to 0.24 V after six scans for InSe-FET in the top liquid gate working mode. Even though the threshold voltage shifts in the liquid gate working mode, the devices fail quickly after six repeated scans. Cyclic tests indicate that the InSe-FET working in the back gate mode with a liquid channel has the best stability under gate electrical stress and is appropriate for biosensing.

**Figure 4:**
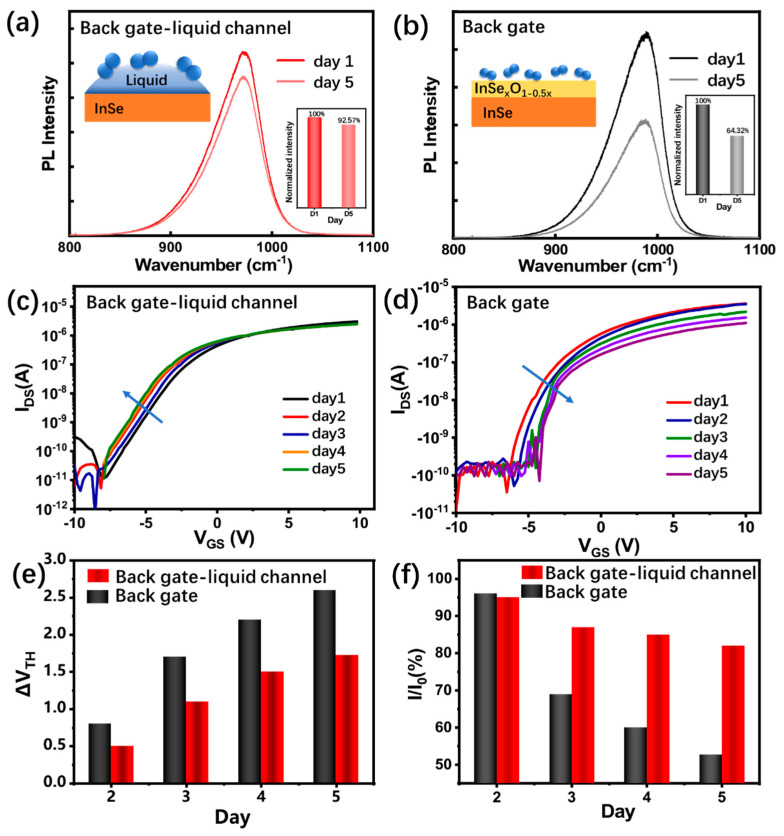
PL spectra of InSe before and after storage for five days in (**a**) liquid and (**b**) atmosphere. Transfer curves before and after storage for five days in (**c**) air and (**d**) liquid. (**e**) The threshold voltage shift and (**f**) the output current change at V_GS_ = 10 V for the InSe-FETs over storage time.

In order to further characterize the stability of the InSe-FET biosensor, the storage stability is tested. The InSe-FETs are stored in atmosphere and liquid, respectively. Figure 4a,b shows the changes in PL spectra after five days. The peak intensity of the InSe immersed in the 0.1×PBS solution declined by 7.43% after five days, while that of the InSe exposed to atmosphere declined by 35.68%. The decrease in the PL peak intensity is probably due to the formation of InSe oxides [34]. The InSe in the atmosphere is exposed to an environment containing both oxygen and water, subjected to oxidation by oxygen. In the liquid channel, the oxidation process is significantly slowed due to the extremely low dissolved oxygen in the liquid. Figure 4c,d shows that the transfer curves shift less in the InSe-FET with the liquid channel than they do with dry channel during the back gate working mode after being stored for five days. When the FET channel is filled with liquid, the threshold voltage shift increases to 1.72 V, and when the channel is exposed to the atmosphere, the threshold voltage shift decreases to 2.59 V. The output current also shows faster degradation for the InSe-FET exposed to the atmosphere than that filled with liquid, as shown in Figure 4f. The electrical properties’ degradation during storage when exposed to air is probably because of the decrease in the intrinsic carrier concentration due to the occurrence of oxidation reactions, leading to the conversion of the material from InSe to InSe_x_O_y_ [24]. The liquid channel protects the damage of InSe from oxidation, and a negative shift in V_TH_ suggests that the InSe is n-doped by ionic adsorbates in liquid.

### 3.4. Subsection

**Figure 5:**
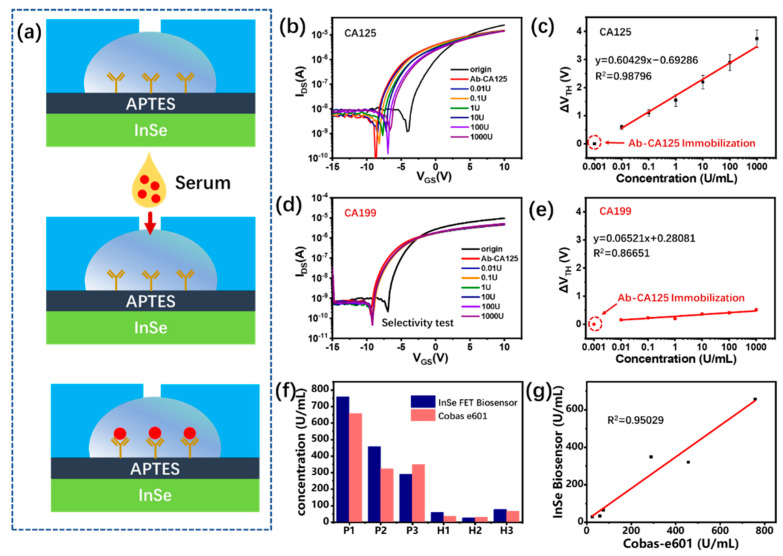
(**a**) Schematic diagram of InSe-FET biosensor for biomarker detection. (**b**) Transfer characteristic responses to target CA125 antigen in different concentrations. (**c**) Quantitative relationship between threshold voltage shift and antigen CA125 concentration. (**d**) Transfer characteristics responses to the non-target antigen CA199, and (**e**) threshold voltage shift corresponding to different concentrations of antigen CA199. (**f**) Comparison of the results of antigen CA125 detection by InSe-FET biosensor and Cobas Roche electroluminescence assay on serum samples from breast cancer patients and healthy individuals. P1~3 are serum samples from three breast cancer patients, and H1~3 are serum samples from three healthy people. (**g**) Correlation of the results of the proposed InSe-FET biosensor with those of the Cobas e601 electroluminescence device.

The specific detection of the cancer marker CA125 is based on the specific binding of antigen–antibody. As shown in Figure 5a, before the detection process, the CA125 capture antibody is immobilized on the InSe channel, and a large number of amino and carboxyl residues on the antibody molecules present charges in the liquid and induce a negative shift in the threshold voltage, as shown in Figure 5b. It is important to note that the scattering effect on the channel carriers induced by the target antigen is limited because of its larger molecule size. The field-effect mobility response for target CA125 is less than 5% and does not show a clear linear relationship with concentration, as shown in Figure S8. Since there is not a dielectric layer between the antibody and the channel material surface, the InSe channel electrons can be directly induced by the antibody, which can be explained from ΔV_TH_ = −Q_F_, where Q_F_ represents the effective charges that can induce the change in the conductivity of the InSe channel [35]. In the meantime, the field-effect mobility drops from 425 cm^2^/V^−1^s^−1^ to 102 cm^2^/V^−1^s^−1^ after the antibody immobilization of InSe, because the charge in direct contact with the surface introduces the Coulomb scattering centers, and the channel electrons are affected by the scattering, which results in a decrease in the field-effect mobility. Once the target sample is loaded into the microfluidic channel, the antigen CA125 is bonded with anti-CA125 and these charges decrease, inducing a positive shift in threshold voltage. The antigen CA125 neutralizes a portion of the antibody-induced gate voltage effect, which is equivalent to applying a small voltage to the InSe surface, visualized as a positive shift in the threshold voltage, and the positive drift of threshold voltage increases with the increasing antigen concentration. Figure 5c shows the linear relationship between the threshold voltage shift and antigen CA125 concentration in the semi-log scale with a detectable range of 0.01–1000 U/mL. Each concentration is tested three times using the same batch devices, and these devices present a standard deviation of 9.05%, as shown in Figure S9. In addition, CA125 can be detected at concentrations as low as 0.01 U/mL, which are much lower those previously reported (0.1 U/mL) [21]. Meanwhile, a comparison of information, such as the detection limits and detection times, between this study and other methods is shown in Table S1. The major contributing factor for the ultralow detection concentration is the ultrasensitive InSe scattering of carriers with small mass and the simple channel treatment. Unlike previously reported protein assays, APTES is used to immobilize antibodies on the surface of InSe without any additional dielectric layer, which greatly improves the ability of the target molecules to modulate the channel current. In addition, the excellent stability of InSe-FETs in liquid is also favorable for the low detection limit.

The specificity of CA125 detection is performed by loading different concentrations of CA199 to the CA125 antibody immobilized channel. Figure 5d shows the negligible response of the InSe-FET to loaded CA199, which demonstrates a nonspecific reaction of CA199 to the antibody CA125. To confirm the ability of the InSe-FET biosensor to detect clinical samples, we collected and tested serum samples from three breast cancer patients and three healthy volunteers. The detected transfer curves and the derived ΔV_TH_ histogram are shown in Figure S10, which presents a much larger transfer curve shift and threshold voltage change in the patients’ serum versus the healthy people’s serum. According to the linear relationship in Figure 5c, the detected concentration of CA125 is derived and shown in Figure 5f in comparison to the results detected by Cobas e 601 from Roche. The proposed InSe-FET biosensor presents a high correlation of R^2^ > 0.95 with commercial Cobas e 601. In addition, the proposed InSe-FET biosensor presents a lower detection limit and rapid detection speed than other representative biosensors, as listed in Table S1. The detection performance indicates the promising application of the proposed InSe-FET biosensor.

## 4. Conclusions

In summary, we tested an InSe-FET biosensor to perform the ultrasensitive, specific, fast, and label-free detection of the breast cancer biomarker CA125. Through systematic experiments and an analysis of InSe-FETs’ electrical characteristics and stability in different working modes, we reveal that the back gate working mode is favorable for conducting biomolecules with filled liquid in the InSe channel. It also indicates that the liquid gate electrode is not suitable for a reactive InSe-based FET biosensor. The proposed biosensor is capable of detecting an ultra-large range of the antigen CA125 from 0.01 to 1000 U/mL with a standard error under 8.78%. The detection of clinical samples has shown that InSe-FET biosensors hold great promise for practical applications, such as the early diagnosis and prognosis of cancer, the study of the pathogenesis of major diseases, and the real-time monitoring of health.

## Figures and Tables

**Table 1:** Material information of detection regents.

Name	Function	Host/Source	Type	Subclass	Purity
CA125	Antibody	Mouse	Monoclonal	IgG1	>90%
	Protein	Mouse	Monoclonal	IgG1	>95%
CA199	Protein	Mouse	Monoclonal	IgG1	>90%

## Data Availability

Not applicable.

## Supplementary Materials

The references in Supplementary Materials were cited in Refs. [36,37,38]. The following supporting information can be downloaded at: https://www.mdpi.com/article/10.3390/bios13020193/s1, Figure S1: Fabrication process of InSe-field transistor biosensor; Figure S2: Typical I_DS_ –V_DS_ curves at 300K for the InSe FET. The device exhibits linear output characteristics at all the temperatures measured.; Figure S3: (a) Real InSe-FET biosensor and detection schematic. (b) Top view of the optical microscope of the biosensor. Figure S4: Comparison of InSe-FET hysteresis using SiO_2_ (a) and HfO_2_/SiO_2_¬ (b) substrates. Figure S5: Surface morphology of (d) bare InSe and (e) APTES modified InSe characterized by AFM. Figure S6: Raman spectra of anti-CA125 on (a) bare InSe and (b) APTES modified InSe, (c) Raman spectra mapping. Figure S7: (a,b) show the distribution of channel electrons after applying gate pressure to the liquid-gate and back-gate FETs, respectively. (c) Schematic diagram of the liquid gate regulated trench inversion layer in the device on state. Figure S8: (a) Evolution of linear coordinate system transfer curve with CA125 concentration. Evolution of mobility with CA125 concentration. Figure S9: Detection curves of the three independent sensors (a), (b), and (c) for the same concentration of CA125, and (d) the threshold voltage shift of the three independent devices. Figure S10: (a) Serum samples from patients and healthy individuals tested using three devices. The threshold voltage offsets extracted for the three patient and healthy human serum samples are shown in (b). Table S1: The detection performance of InSe-FET biosensor and other representative biosensors.
