# Supplementary material for: Highly Stable InSe-FET Biosensor for Ultra-Sensitive Detection of Breast Cancer Biomarker CA125
Source: Biosensors (Basel). 2023 Jan 28;13(2):193. doi: 10.3390/bios13020193 (PMC9954013; doi:10.3390/bios13020193)
Supplement: Supplementary file 1 [file biosensors-13-00193-s001.zip › biosensors-2097841-supplementary.pdf]

Supplementary Materials

Highly Stable InSe-FET Biosensor for Ultra-Sensitive Detection of Breast Cancer Biomarker CA125

Hao Ji [1], Zhenhua Wang [1], Shun Wang [1], Chao Wang [1], Kai Zhang [1], Yu Zhang [1,*] and Lin Han [1,2,3,*]

[1] Institute of Marine Science and Technology, Shandong University, Qingdao 266237, China
[2] Shenzhen Research Institute of Shandong University, Shenzhen 518057, China
[3] Shandong Engineering Research Center of Biomarker and Artificial Intelligence Application, Ji'nan 250100, China
* Correspondence: yuzhang@sdu.edu.cn (Y.Z.); hanlin@sdu.cn (L.H.)

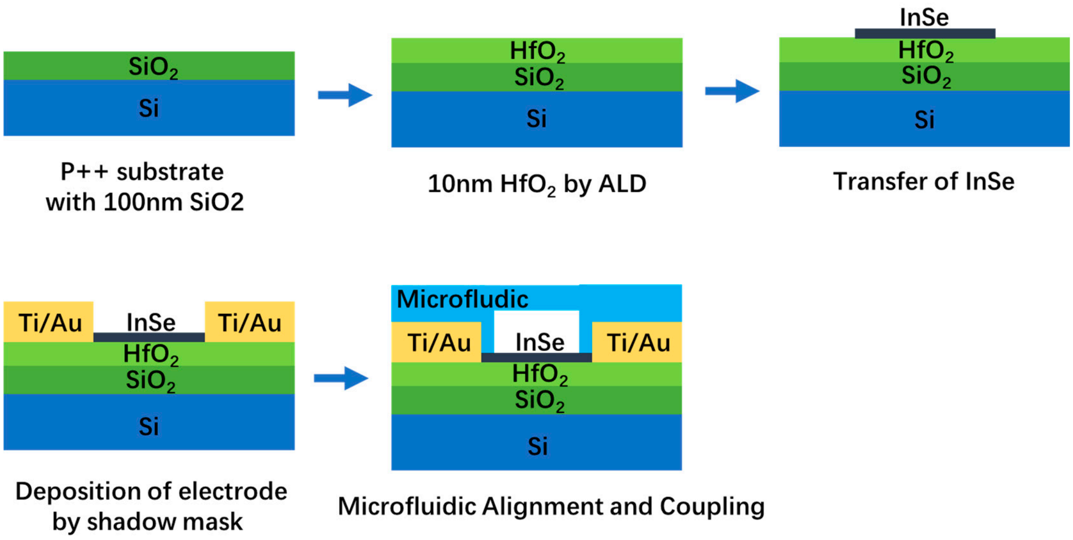

Figure S1. Fabrication process of InSe-field transistor biosensor.

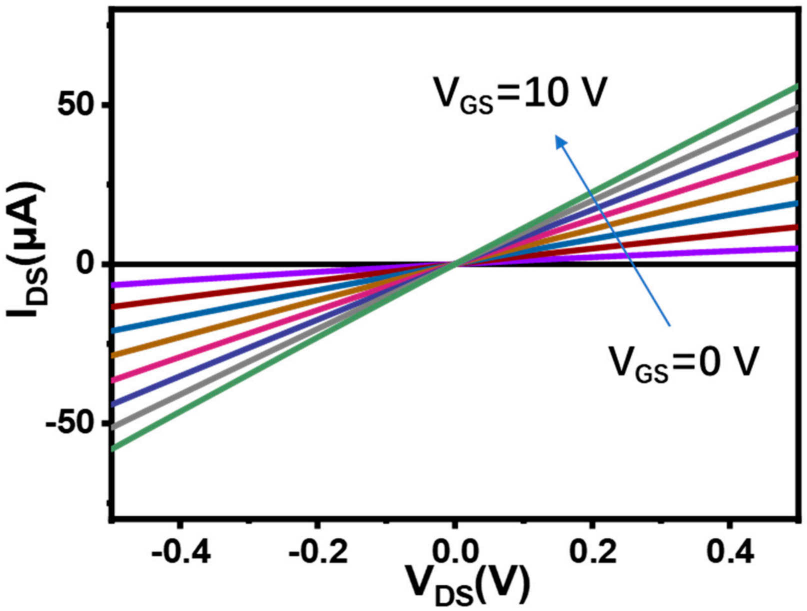

Figure S2. Typical I_{DS} –V_{DS} curves at 300K for the InSe FET. The device exhibits linear output characteristics at all the temperatures measured.

Detection is performed by applying voltage to the back gate and drilling holes in the microfluidic channel for sample addition and detection.

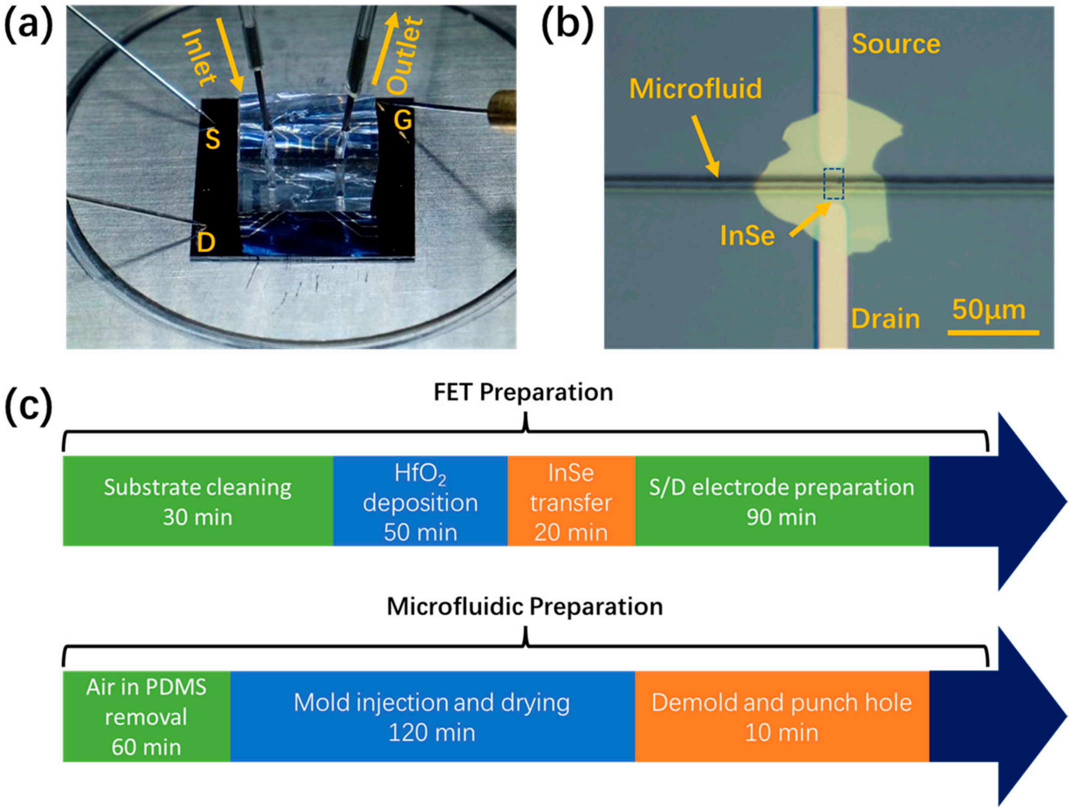

Figure S3. (a) Real InSe-FET biosensor and detection schematic. (b) Top view of the optical microscope of the biosensor.

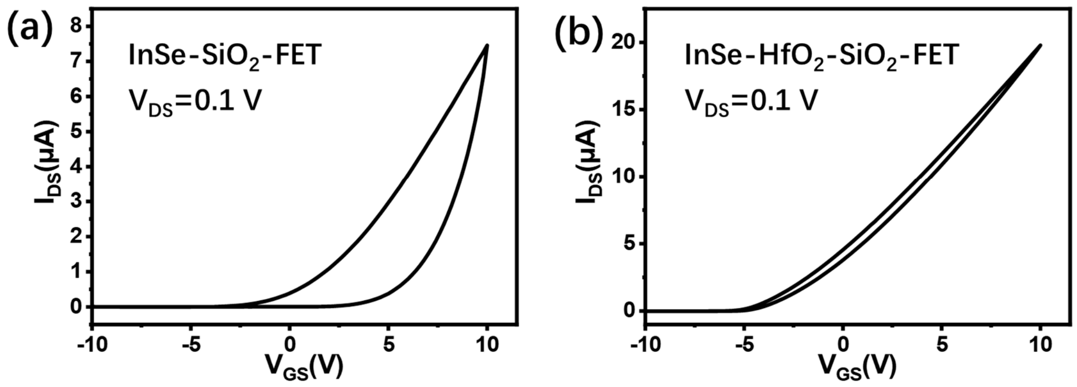

Figure S4. Comparison of InSe-FET hysteresis using SiO_2 (a) and HfO_2/SiO_2 (b) substrates.

The AFM system was used to measure surface morphology of InSe before and after immobilized with APTES. It can be seen from Figure S6a that the surface roughness of APTES modified InSe is higher, with a value of 0.5062 compared with bare InSe, which means the success in functionalization.

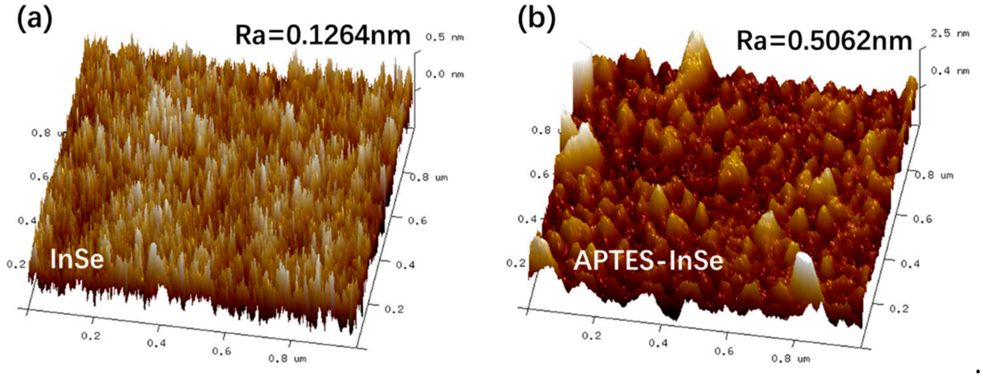

Figure S5. Surface morphology of (d) bare InSe and (e) APTES modified InSe characterized by AFM.

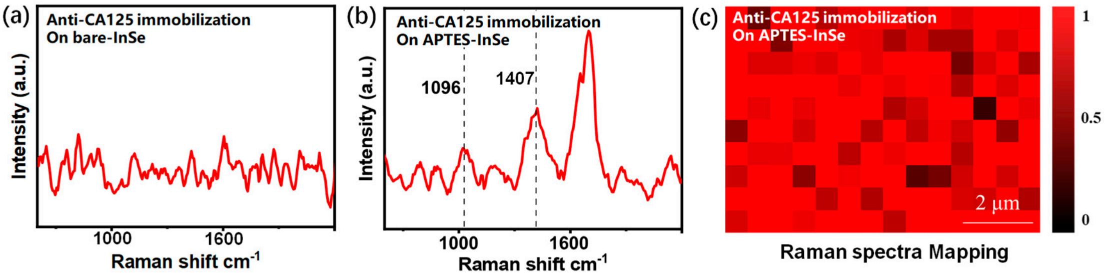

Figure S6. Raman spectra of anti-CA125 on (a) bare InSe and (b) APTES modified InSe, (c) Raman spectra mapping.

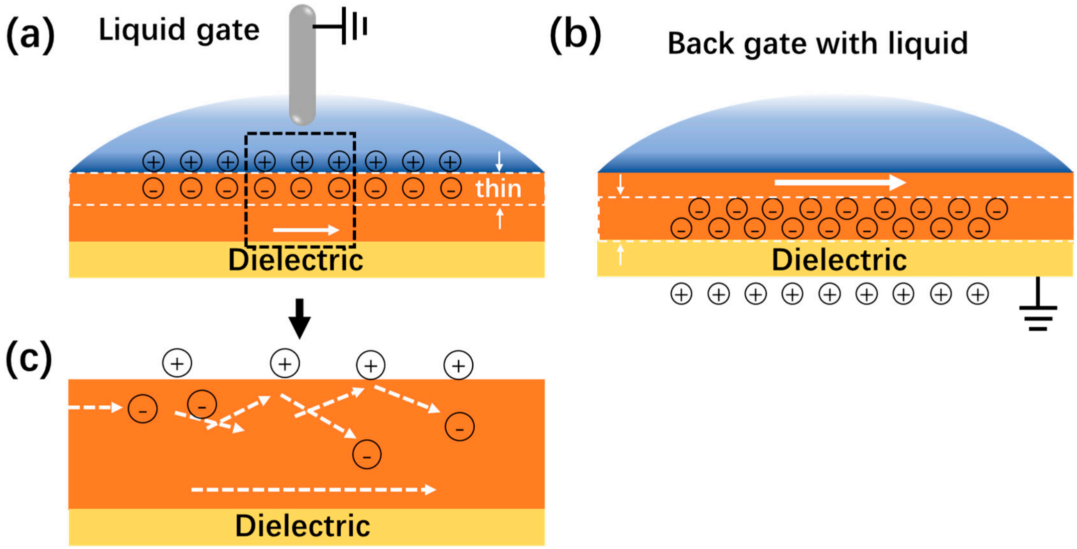

.

Figure S7. (a,b) show the distribution of channel electrons after applying gate pressure to the liquid-gate and back-gate FETs, respectively. (c) Schematic diagram of the liquid gate regulated trench inversion layer in the device on state.

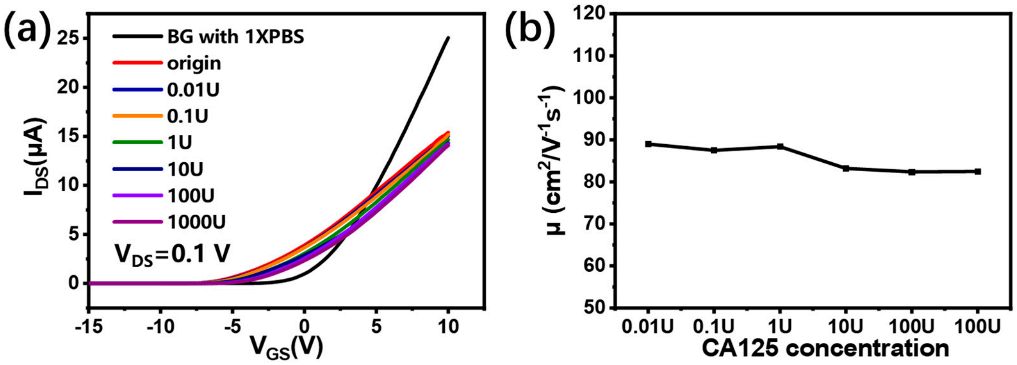

Figure S8. (a) Evolution of linear coordinate system transfer curve with CA125 concentration. Evolution of mobility with CA125 concentration.

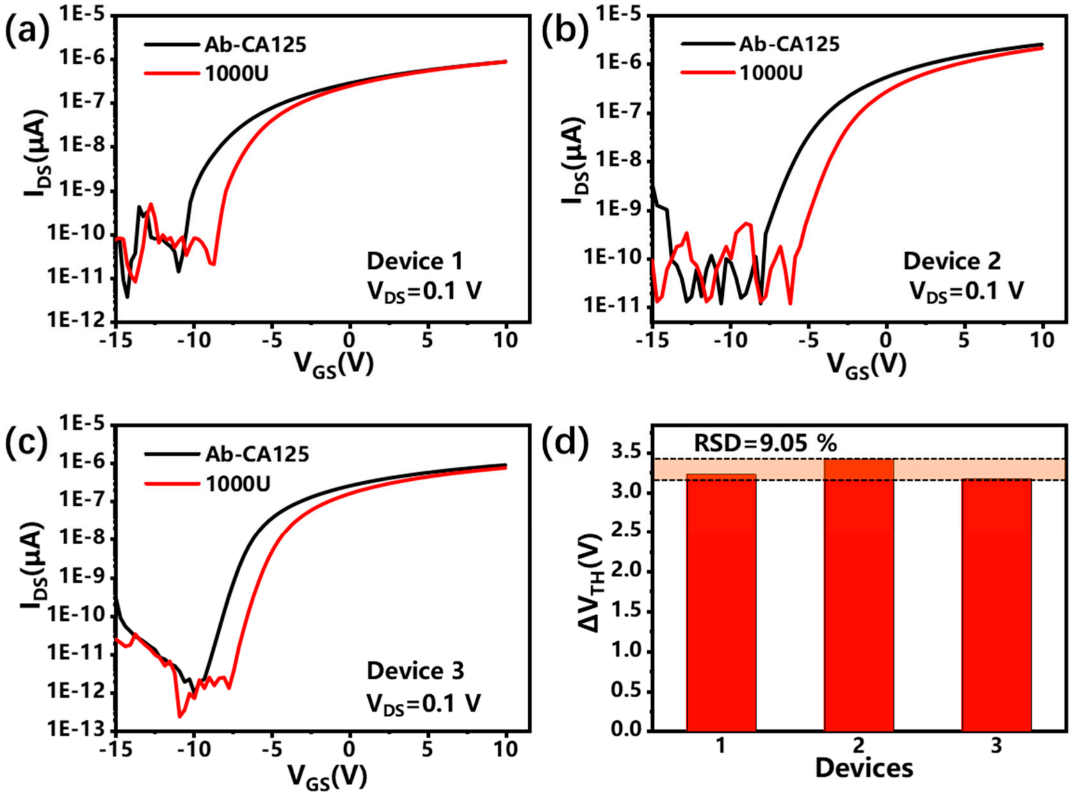

Figure S9. Detection curves of the three independent sensors (a), (b), and (c) for the same concentration of CA125, and (d) the threshold voltage shift of the three independent devices.

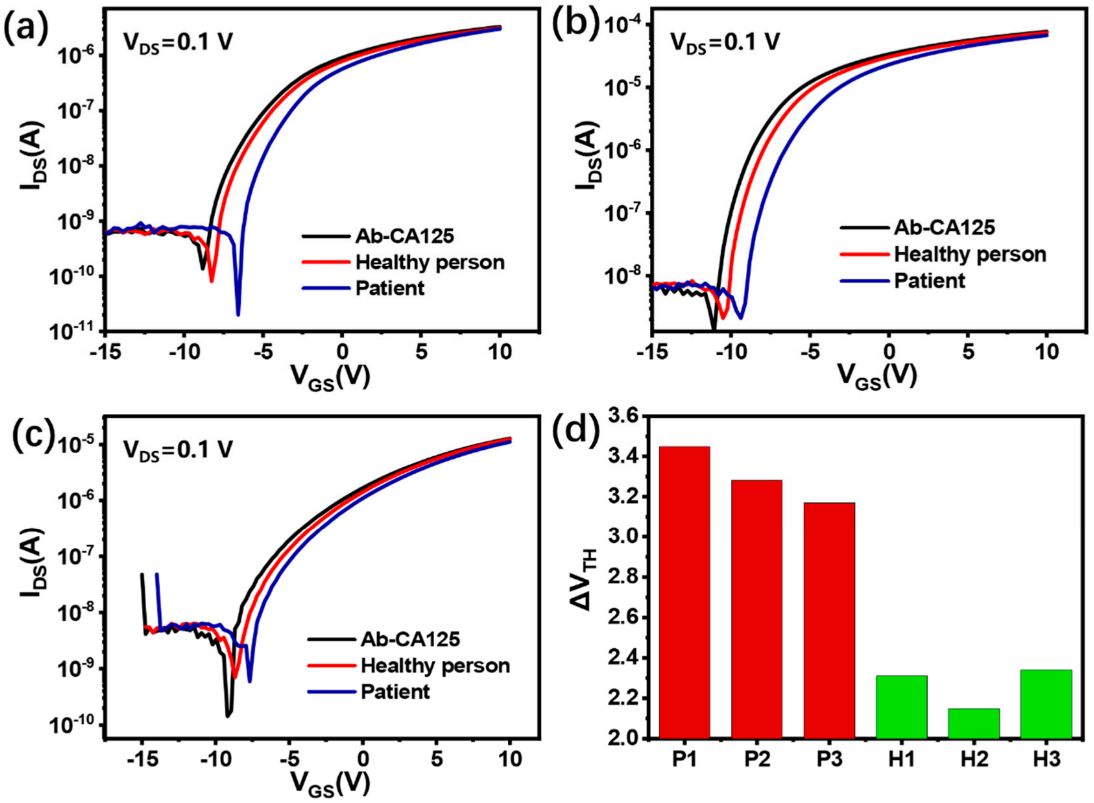

Figure S10. (a) Serum samples from patients and healthy individuals tested using three devices. The threshold voltage offsets extracted for the three patient and healthy human serum samples are shown in (b).

Table S1. The detection performance of InSe-FET biosensor and other representative biosensors.

Detection method	Biomarker	Detection limit	detection time	Ref.
Microfluidic/ electrochemical	CA199	10.75U/mL	35min	1
multiplexed detection	CA199	6U/mL	2h	2
	CA125	8.5U/mL	2h	
GQDs microfluidic	CA125	0.1U/mL	40min	3
InSe-FET biosensor	CA125	0.01U/mL	30min	This work

Refreences

1. Y. Xie, X. Zhi, H. Su, K. Wang, Z. Yan, N. He, J. Zhang, D. Chen, D. Cui, A novel electrochemical microfluidic chip combined with multiple biomarkers forearly diagnosis of gastric cancer, Nanoscale Res Lett 10 (1) (2015) 477.
2. X.-H. Li, W.-M. Sun, J. Wu, Y. Gao, J.-H. Chen, M. Chen, Q.-S. Ou, An ultrasensitive fluorescence aptasensor for carcino-embryonic antigen detection based on fluorescence resonance energy transfer from up conversion phosphors to Au nanoparticles, Anal. Methods 10 (13) (2018) 1552e1559.
3. M. Terenghi, L. Elviri, M. Careri, A. Mangia, R. Lobinski, Multiplexed determination of protein biomarkers using metal-tagged antibodies and size exclusion Chromatography Inductively coupled plasma mass spectrometry, Anal. Chem. 81 (22) (2009) 9440e9448. C. Wang, Y. Zhang, W. Tang et al. Analytica Chimica Acta 1178 (2021) 33879110
